# Adolescents’ Well-being While Using a Mobile Artificial Intelligence–Powered Acceptance Commitment Therapy Tool: Evidence From a Longitudinal Study

**DOI:** 10.2196/38171

**Published:** 2022-11-29

**Authors:** Dana Vertsberger, Navot Naor, Mirène Winsberg

**Affiliations:** 1 Stanford University Stanford, CA United States; 2 Kai.ai Haifa Israel

**Keywords:** well-being, adolescents, chatbots, conversational agents, mental health, mobile mental health, automated, support, self-management, self-help, smartphone, psychology, intervention, psychological, therapy, acceptance, commitment, engagement

## Abstract

**Background:**

Adolescence is a critical developmental period to prevent and treat the emergence of mental health problems. Smartphone-based conversational agents can deliver psychologically driven intervention and support, thus increasing psychological well-being over time.

**Objective:**

The objective of the study was to test the potential of an automated conversational agent named Kai.ai to deliver a self-help program based on Acceptance Commitment Therapy tools for adolescents, aimed to increase their well-being.

**Methods:**

Participants were 10,387 adolescents, aged 14-18 years, who used Kai.ai on one of the top messaging apps (eg, iMessage and WhatsApp). Users’ well-being levels were assessed between 2 and 5 times using the 5-item World Health Organization Well-being Index questionnaire over their engagement with the service.

**Results:**

Users engaged with the conversational agent an average of 45.39 (SD 46.77) days. The average well-being score at time point 1 was 39.28 (SD 18.17), indicating that, on average, users experienced reduced well-being. Latent growth curve modeling indicated that participants’ well-being significantly increased over time (β=2.49; *P*<.001) and reached a clinically acceptable well-being average score (above 50).

**Conclusions:**

Mobile-based conversational agents have the potential to deliver engaging and effective Acceptance Commitment Therapy interventions.

## Introduction

Adolescence is a developmental period that is filled with changes: changes to one’s body, in one’s social environment, and even to one’s mind [1]. It is also a crucial period for mental, social, and emotional well-being, which is characterized with an increased risk to develop mental health problems, such as anxiety, depression, substance abuse, and eating disorders [1]. According to the Centers for Disease Control and Prevention, more than 10% of adolescents aged 12-17 years experience anxiety, almost 8% experience behavior disorders, and 6% experience depression. These problems tend to continue during adulthood, especially when left untreated, and impact not only those who experience it but also those around them, as well as society as a whole. For example, lost productivity due to anxiety and depression is estimated to cost the global economy US $1 trillion each year [2]. The increased risk in developing mental illnesses during adolescence marks it as a crucial period for prevention, as well as treatment, and emphasizes the need for accessible and customized mental health tools aimed at decreasing adolescents’ ill-being and increasing their well-being. In this study, we focused on adolescents’ well-being and followed their well-being during a time in which they used a digital, artificial intelligence (AI)–powered, personal companion designed to promote well-being and mental health.

Well-being usually refers to (1) a desirable state of satisfaction; (2) the presence of positive affect (eg, happiness); and (3) the absence of negative affect [3,4]. Well-being does not refer to a specific moment but rather a continuous state [5]. Previous research that mainly focused on adult populations have shown that psychological well-being is associated with health and long-term adjustment [6]. Specifically, higher levels of well-being were found to be associated with fewer illnesses, increased life expectancy, and healthier behavior [6]. In comparison, lower levels of well-being were found to be associated with higher levels of depression, hopelessness, and suicidal intent [7], as well as actual suicide attempts [8,9].

The few longitudinal studies that did focus on adolescents’ well-being tend to find that adolescents’ well-being decreased over time. Specifically, it was found that well-being, which was often measured by satisfaction with school, family, friends, schoolwork, appearance, and life as a whole, showed weak stability over time [10] and that girls are at greater risk of a decrease in their well-being over time than boys [10-12]. Another study did not find a significant change over time in psychological well-being but did find that girls reported lower levels of well-being than boys [13]. Taken together, these studies suggest that adolescent girls are at a greater risk to experience reduced well-being than boys.

Studies on adult populations suggest that different interventions can improve well-being, compared to control groups who did not receive interventions or were in delayed intervention groups. For example, an intervention based on Acceptance and Commitment Therapy (ACT), which focuses on cognitive diffusion, psychological flexibility, mindfulness, and values clarification, was found to be associated with greater well-being posttreatment, as well as at the 3-month follow-up, compared to the control group who did not receive the intervention [14]. Another study tested the effectiveness of the “My Coping Plan” app in improving mental health and coping [15]. The “My Coping Plan” approach focuses on normalizing unpleasant emotions and coping as universal human experiences, both for those with and without mental illness, and encourages professional help–seeking as a healthy coping strategy when personal coping strategies are ineffective. It was found that participants in the intervention group reported improved well-being, compared to control group, over the 1-month period of using the app [15]. Finally, a study that tested the effect of mindfulness-based therapy versus a waitlist group among patients with breast cancer showed a significant increase in well-being in the experimental group at both 8- and 12-week assessments compared to the control group [16].

One promising avenue for delivering interventions, especially for adolescents, is through their smartphones. The use of smartphones and their different apps have been highly integrated in almost everyone’s everyday lives. Almost 50% of 11-year-olds in the United States have a mobile phone, with this number reaching 85% among 14-year-olds [17]. On average, US adolescents aged 13-18 years engage with their mobile phone for more than 3 hours every day [18], making it a highly accessible and easy-to-use tool for presenting a mental health intervention. Indeed, recent years have seen an increase in the development of various mental mobile health (mHealth) interventions apps [19]. These mental mHealth interventions are either aimed at complementing traditional mental health treatments or providing mental health support to those who are unable to receive quality mental health services—for example, due to long waiting lists [19]—and were found to be beneficial to adult populations [19-21].

There are many possibilities for delivering mental mHealth interventions, such as web-based therapists, conversational interfaces (such as Amazon’s Alexa), and delivering information. Another prospective approach, especially for adolescents, is by implementing a text-based approach. Using a text-based approach could be an easy and sustainable way to keep adolescents engaged with the process of the intervention. Thus, in this study, we tested whether adolescents’ well-being improved while using a commercially developed text-based conversational agent named Kai.ai.

Kai.ai is an AI-powered, personal companion designed to promote well-being and mental health by initiating daily conversations and presenting short and simple exercises to users and is used within an instant messenger app (eg, iMessage and WhatsApp). The intervention delivered by Kai.ai is mainly based on ACT protocols and tools adapted from positive psychology theories. As previously described, ACT aims to improve cognitive flexibility, as well as coping with challenging experiences, by focusing on cognitive diffusion, practicing mindfulness, and reflection on one’s values [22,23]. Kai.ai delivers the ACT intervention using an AI conversational bot that facilitates users in creating and enhancing habits for healthy living and resilience [24]. Kai.ai interacts with users throughout the day and leverages their responses to deliver a tailored ACT skilled coaching. The main advantages of Kai.ai are that (1) it integrates seamlessly with all the top messaging apps (iMessage, WhatsApp, Discord, Telegram, etc), making it accessible and easy to use; (2) it can reach adolescents who would otherwise not seek support; (3) it contacts the adolescents but also responds to adolescents when they initiate the conversation; (4) it is available 24/7; (5) it is free; and (6) it is anonymous.

In this study, we followed adolescents who voluntarily chose to join and interact with Kai.ai. At several time points during the period in which they interacted with Kai.ai, they were asked about their well-being. We hypothesized that their well-being would improve while using Kai.ai.

## Methods

### Participants

The initial sample included 43,237 adolescents from the United States, aged 14-18 years, who had a smartphone with either an iOS or Android operating system and freely chose to interact with Kai.ai through common messaging apps. We advertised Kai.ai in platforms that are frequently used by adolescents such as Instagram and Snapchat. Within these platforms, the advertisements were specifically shown to adolescents aged 14-18 years, and especially girls, according to the information they entered during the registration to each platform. Users are presented with an ad inviting them to test how happy they are. When clicking the ad, users are taken to Kai.ai’s landing page. The final sample included 10,387 participants who answered the questionnaires more than once. The onboarding process for the use of Kai.ai does not ask the user to report their actual age or gender; thus, it did not enable us to report this information.

### Procedure

As part of the joining process to Kai.ai (in the onboarding process), all users were asked, but were not obligated, to complete different questions and questionnaires to assess their needs. Subsequently, users were prompted once every 6 weeks to answer these questionaries once again, to monitor their mental progress; however, they could have answered whenever it was convenient for them. These prompts were also optional, and users were not required to answer the questionnaires to continue using Kai.ai. Participants in this study completed the 5-item World Health Organization Well-being Index (WHO-5) questionnaire between 2 and 5 times, between February 2020 and January 2022. The study was based on anonymized data gathered during the engagement with the service.

### Ethics Approval

This study was approved by the WCG Institutional Review board (approval #1-1504102-1) and determined as not human subject research.

### Intervention

#### Kai.ai

During the onboarding process, the users are made clear that Kai—the name of AI in the program—is not a real person and has been built by clinical psychologists, coaches, and engineers. Users are being informed that Kai will reach out to them and will send them daily practices, techniques, and insights, but they are also encouraged to reach out to Kai whenever they need. Kai.ai initiates between 1 and 3 daily interactions with the users, usually in the morning, at noon, and in the evening.

Each daily interaction that is initiated by Kai.ai begins with a greeting (ie, “good morning” or “good evening”) and an inspiration quote (eg, “Many people will walk in and out of your life, but only true friends will leave footprints in your heart,” Eleanor Roosevelt), which are then followed by a short exercise (described below) related to ACT. Users can also initiate an interaction with Kai whenever they please, or when Kai initiates the interaction, they can direct it to whatever topic they wish. When Kai recognizes that participants are in some sort of distress, the bot switches off and a trained companion, who is practiced in giving support, goes on and encourages users to turn to a someone close to them for support or use available hotlines near them (ie, presents them with a list of available possibilities).

#### Process-Oriented Features

In addition to interacting with Kai, Kai.ai also presents users with different exercises to promote better mental health. These exercises are described below.

##### Gratitude

The aim of this exercise is to help users to develop flexible thinking patterns, balance negative biases, and develop a positive view over their lives. Each day, users are prompted to think about the things they are grateful for and share them with Kai. The system saves their responses, and they can view them whenever they want.

##### Learning

The aim of this exercise is to help users to adopt a routine of reflection and journaling to help them achieve a more centered, grounded, joyous, and purposeful state of mind. Users are prompted to focus on the lessons they can learn from their experiences. In addition, to reduce stress and anxiety, users are guided to focus on a single task they have instead of a long to-do list.

##### Breathing

The breathing exercises are meant to reduce stress and anxiety by ensuring a better flow of oxygen to the body through the operation of the parasympathetic nervous system [25]. Initially, users are taught in a relaxed state of mind with the aim that with continuous practice, the exercises will become a tool that can be enacted while the users feel distressed. The exercises teach users how to breathe through their noses, using their diaphragm, and note their posture.

##### Mindfulness

The mindfulness exercises help the users become aware of the present moment without being judgmental toward themselves. The mindfulness exercises are audio-based and help users practice the art of observing and visualizing thoughts, emotions, and body sensations as they arise. By practicing these exercises, users can benefit by letting go of any repeated unwanted thoughts, increase self-awareness and self-compassion, as well as reduce tension and stress.

##### ACT Training

The ACT training aims to develop psychological flexibility and diffusion of thoughts, emotions, and behaviors and accept the challenging moments in our lives that contain negative emotions, as well as unfold the users’ values and help them commit to the values. It teaches users to treat pain and discomfort as facts of life that can be used for personal growth through a process of acceptance and validation [26]. ACT exercises are also presented as audio, in which their aim is to guide the users in observing and accepting their current and past thoughts and emotions, despite the discomfort they might elicit. These ACT exercises help build resilience, gain control over thoughts and emotions, and assist in building coping strategies when facing difficult moments.

##### Positive Psychology

These exercises aim to decrease negativity bias and increase positivity in users’ lives and are also audio-based. They help connect positive intentions to the users themselves and assist in enhancing self-compassion.

### Measures: Well-being

Users’ well-being was assessed using the WHO-5 Well-being Index, which is a brief 5-item self-reported measure [27]. Participants were asked to report their experiences in the past 2 weeks (eg, “I have felt cheerful and in good spirits”) on a 6-point Likert scale, ranging from 0 (at no time) to 5 (all of the time). The raw well-being score theoretically ranges from 0 (absence of well-being) to 25 (maximal well-being). However, as scales measuring health-related quality of life are typically translated to a percentage-based scale, ranging from 0 to 100, it is recommended to multiply the raw score by 4. A score below 50% reflects poor well-being, and a 10-point change in the translated score is seen as clinically significant [28]. The WHO-5 has shown both clinical and psychometric validity (for a systematic review, see Topp et al [28]) and has been previously integrated in mental health– and physical health–related mobile apps [29-31]. The WHO-5 was also found to be reliable for assessing children and adolescents’ well-being [32-34].

### Statistical Approach

**Figure 1:**
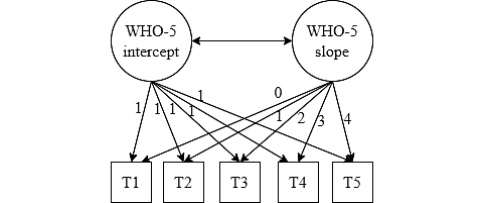
An illustration of the latent growth curve model. T: time point; WHO-5: 5-item World Health Organization Well-being Index.

We first conducted a 1-way ANOVA to test for differences in the WHO-5 baseline scores between participants according to the number of times they have answered the questionnaire. In this analysis, we also considered those who answered only once, to test whether there were differences in well-being between those who chose to answer only once and those who continued to answer the questionnaire. Next, we described the descriptive statistics of the sample. Finally, to account for the structure of data, in which we had several time points of assessment for each user (between 2 and 5), we conducted latent growth curve modeling, in which we examined the change in the WHO-5 assessment over time. We first conducted an intercept-only model, in which the intercept variance was constrained to 0, so we only assessed the mean. In the second model, we conducted a random-intercept model and allowed users to differ in their starting point. In the third model, we added a random slope (ie, users are changing in different ways) but set the average slope to 0. Finally, in the fourth model, we estimated the real slope. After each step, we estimated the fit of the model to test whether adding each component improved the model. Figure 1 is an illustration for the final model. The analysis was conducted using the *lavaan* package in R statistical software (version 3.5.1; R Foundation for Statistical Computing) [35].

## Results

First, we tested for differences in WHO-5 baseline scores between all participants according to the number of times they have answered the questionnaire, using the *aov* function in R statistical software [36]. No significant difference was found between the groups (*F*_4,43,232_=0.35; *P*=.84).

On average, users interacted with Kai.ai for 45.39 (SD 46.77; range 2-634) days. Table 1 describes the averages of the WHO-5 assessment at each time point (T). The average well-being score at T1 was below 50 (mean 39.28, SD 18.15), indicating that, on average, users experienced reduced well-being [28]. However, the average score increased over time, reaching an average of 53.63 (SD 21.32) in T5, 85 days after the first assessment (approximately 2.5 months). The average number of days that have passed between each assessment increased over time, with the average number of days being 25.80 days between T1 and T2 and 36.62 days between T4 and T5.

**Figure 2:**
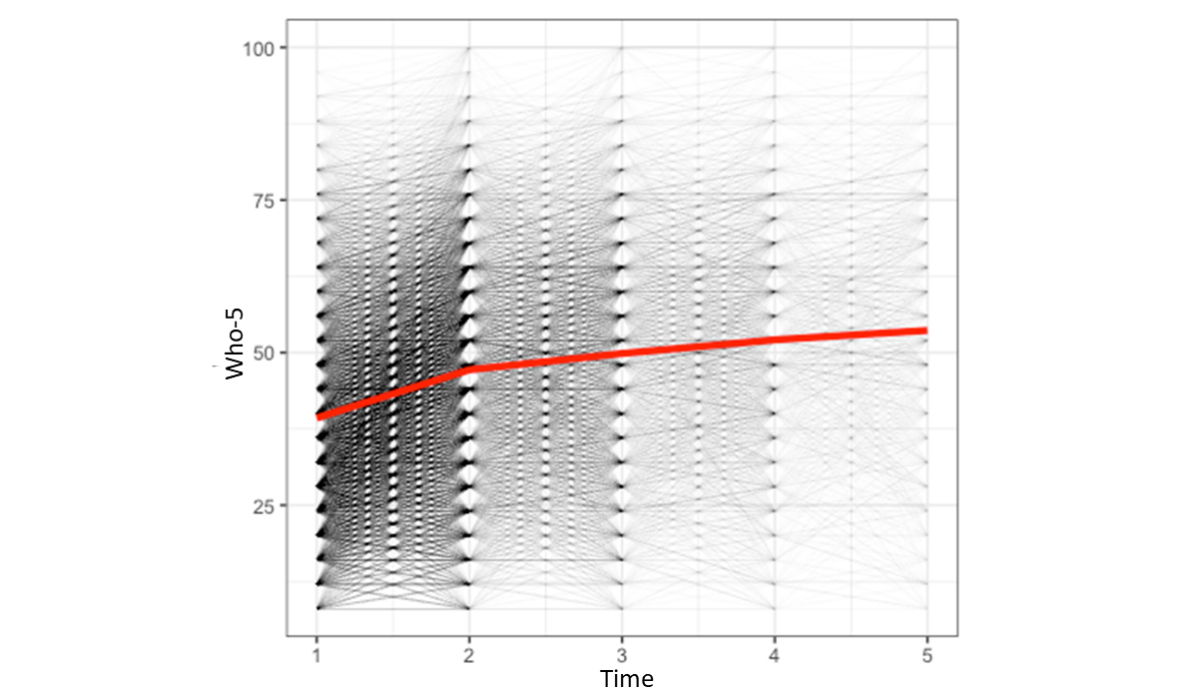
Plot of the change in the WHO-5 scores across time. WHO-5: 5-item World Health Organization Well-being Index.

The results of the latent growth curve modeling, in which we assessed the difference in the WHO-5 assessments over time, are presented in Table 2. As can be seen in Table 2, the model fit improved from the first model to the last model, with the fourth model showing the best goodness of fit. As can be seen in the fourth model, participants’ well-being significantly increased over time (β=2.49; *P*<.001). Figure 2 depicts the change in the WHO-5 over time. However, the negative covariance between the intercept and slope indicates that users who were lower in well-being show a bigger increase in well-being than users who were higher in well-being, as could be expected.

**Table 1:** Number of participants, mean, and SD of the WHO-5^a^ assessments at each time point and average time elapsed between each time point.

T^b^	Participant, n	WHO-5 score, mean (SD)	Average time between T_n_ – T_n + 1_
1	10,387	39.28 (18.17)	
2	10,387	47.18 (19.68)	25.80
3	4801	49.85 (20.15)	25.70
4	2324	52.09 (20.45)	30.81
5	1072	53.64 (21.32)	36.62

^a^WHO-5: 5-item World Health Organization Well-being Index.

^b^T: time point.

**Table 2:** Latent growth curve modeling of the associations between time and users’ engagement and the 5-item World Health Organization Well-being Index.

Model	Intercept (σ^2^)	SE	Slope (σ^2^)	Intercept-slope covariance	Goodness of fit			
					*χ*^2^ (*df*)	RMSEA^a^	SRMR^b^	CFI^c^
Intercept only	50.25 (0)	393.2	N/A^d^	N/A	2884.86 (18)	0.39	0.43	0
Random intercept	50.25 (212.03)	181.70	N/A	N/A	794.52 (17)	0.21	0.14	0.7
Random slope	47.77 (188.68)	142.42	0 (14.22)	0	493.12 (16)	0.17	0.15	0.82
Real slope	45.28 (209.13)	138.13	2.49 (11.04)	–8.33	227.55 (14)	0.12	0.06	0.92

^a^RMSEA: root mean square error of approximation.

^b^SRMR: standardized root mean square residual.

^c^CFI: comparative fit index.

^d^N/A: not applicable.

## Discussion

### Principal Findings

Adolescence is a crucial period for treating and, ideally, preventing mental health problems and increasing well-being: first, to improve the lives of adolescents and second, to decrease the risk for developing mental health problems in adulthood [1]. In this study, we followed the well-being of more than 1000 adolescents from the United States through their interaction with an AI-powered, personal companion named Kai.ai, over a period of 4 months. The results indicated that, on average, adolescents’ well-being increased over time and went from, on average, a poor well-being score to an acceptable well-being score (above 50).

The importance of developing cost-effective, accessible, and engaging mental health interventions lies not only in the obvious benefits they have for adolescents and their families’ well-being but also in the economic impact they may have. For example, the overall annual economic burden of depression among adults in the United States is estimated to be greater than US $326 billion [37], which were mainly accounted for by workplace costs (eg, missed days of work and reduced productivity while at work). Such studies among adolescents are scarce, but it was estimated that the annual societal cost of clinically referred adolescents ranged between US $42-66 million [38]. These costs were mainly attributed to their parents’ loss of productivity and adolescents’ school absence. When adding the economic impacts of other disorders, such as anxiety, these estimates are much higher. Thus, findings ways to efficiently treat, and more importantly prevent, mental health problems and increase well-being should be considered a priority for policy makers, health care providers, and entrepreneurs.

### Limitations

The assessments were made through the Kai.ai platform, but we cannot infer that the improvement of users’ well-being stemmed directly from the use of the service for several reasons. First, since the participants were all users who freely chose to use the service, there was no control group that was followed and studied for the same duration of the study. Therefore, users’ improvement may represent a regression to the mean. Moreover, individuals who freely chose to use Kai.ai, a self-help tool, may have used other self-help tools or apps at the same time. Future studies should conduct a randomized control trial to better understand the effectiveness of the service compared to no intervention. Second, the use of common messaging apps for communicating with the users made it impossible to monitor whether they used the different process-oriented features such as breathing and ACT training. Therefore, we could not test their associations with users’ well-being. Third, as the use of Kai.ai is anonymous, we did not have estimates regarding age, gender, and other demographic variables such as socioeconomic status, which limits the generalizability of the findings.

### Conclusions

These initial results demonstrate the potential of a text-based conversational companion as a cost-effective and accessible tool to improve adolescents’ well-being. Due to the great economic cost for poor well-being and mental health and the decrease in the accessibility of various support systems, partly due to the COVID-19 pandemic, developing efficient interventions should be considered a societal priority. Future studies should test if any of the process-oriented features of Kai.ai are beneficial for improving users’ well-being or if the recognized increase in well-being represents a regression to the mean.

## Abbreviations

ACT: Acceptance Commitment Therapy
AI: artificial intelligence
mHealth: mobile health
T: time point
WHO-5: 5-item World Health Organization Well-being Index
